# Parents and children active together: a randomized trial protocol examining motivational, regulatory, and habitual intervention approaches

**DOI:** 10.1186/s12889-020-09465-z

**Published:** 2020-09-21

**Authors:** Stina J. Grant, Mark R. Beauchamp, Chris M. Blanchard, Valerie Carson, Benjamin Gardner, Darren E. R. Warburton, Ryan E. Rhodes

**Affiliations:** 1 Behavioural Medicine Laboratory, School of Exercise Science, Physical and Health Education University of Victoria, Victoria, Canada; 2 School of Kinesiology, University of British Columbia, Vancouver, Canada; 3 Department of Medicine, Dalhousie University, Room 205 Centre for Clinical Research, 5790 University Avenue, Halifax, Nova Scotia B3H 1V7 Canada; 4 University of Alberta, Edmonton, Canada; 5 King’s College London, London, UK

**Keywords:** Family physical activity, Parent-child co-activity, Habit formation

## Abstract

**Background:**

Regular physical activity (PA) is associated with many health benefits during childhood, and tracks into desirable PA patterns and health profiles in adulthood. Interventions designed to support these behaviours among young children are critical. Family-based interventions focusing on parent-child activities together (i.e., co-activity) among preschool-aged children are warranted. Targeting parental support practices can increase the frequency of co-activity, however interventions must move beyond merely building intention and planning skills for successful maintenance. Interventions designed to increase co-activity habit strength may facilitate the sustainability and thus impact child PA. The purpose of this study is to compare the effects of three intervention conditions designed to increase child PA through co-activity: a standard *education* condition (information about benefits), a planning (action planning, coping planning) + education condition and a habit (context-dependent repetition from prompts and cues) + planning +education condition.

**Methods/design:**

A longitudinal three-arm parallel design randomized trial will compare three conditions over six months. Families are eligible if they have at least one child between 3y and 5y that is not meeting 60mins/day of moderate to vigorous physical activity (MVPA). The primary outcome (child MVPA) is assessed via accelerometry at baseline, six weeks, three months and six months (primary endpoint). Intervention materials targeting co-activity are delivered post baseline assessment, with booster sessions at six weeks and three months. Parental co-activity habit, parent-child co-activity and other behavioural constructs are also assessed via questionnaire at all measurement occasions. As tertiary outcomes, parental PA is measured via accelerometry and co-activity is measured via a Bluetooth-enabled proximity feature. A total of 106 families have been recruited thus far from the Greater Victoria region. The study is ongoing with a minimum target of 150 families and an anticipated recruitment completion date of August 2022.

**Discussion:**

This protocol describes the implementation of a randomized trial evaluating the effectiveness of a *habit formation* group compared with a *planning* group and an *education* only group to increase child PA through targeting parent-child co-activity. This information could prove useful in informing public health initiatives to promote PA among families with preschool-aged children.

**Trial registration:**

This trial was prospectively registered on clinicaltrials.gov in February 2016, identifier NCT03055871.

## Background

Regular physical activity (PA) is associated with a 20–30% risk reduction for over 25 chronic conditions [1]. Unfortunately, many Canadian adults and children do not meet recommended guidelines to optimize these benefits [2, 3]. The antecedents of health risk factors begin in early life, making primary prevention efforts in childhood of paramount importance [4]. To this end, although disease and health complications typically present in adulthood, there is convincing evidence to suggest regular PA among children protects against many negative physical, psychological, social, and cognitive health outcomes [5–8]. Effective interventions aimed at helping preschool-aged children develop PA patterns are critical for long-term population health. Designing such interventions requires an understanding of factors that influence PA of preschool-aged children.

Developing PA practices before formal schooling may be optimal for cementing health behaviours to be carried through the school years and ultimately establishing a pattern for PA over the life-course [9]. Thus, the home is one important environment to target and there remains a need to promote PA within the family unit. Preschool-aged children aged three to 5 years spend considerable time in the care of their parents. Indeed, parents appear to be the ‘gatekeepers’ of PA during family time [10, 11]. Unsurprisingly, parental influence on PA has received considerable research attention [12] and a review of family-based interventions found parent support to be a consistent determinant of child PA [13]. One type of parental support is co-participation, whereby parents facilitate activities in which they can be active with their children. Co-activity is defined herein as any energetic activity that gets parent and child moving together, through which the child is active at a moderate or vigorous intensity. Co-activity has been identified as a crucial factor in the support of PA for preschool-aged children [14]. A recent national survey examining parental beliefs on co-activity found that early childhood was considered the optimal time for co-activity practice [15]. This type of support has numerous advantages: it models an active lifestyle [16], fosters healthy family dynamics though bonding [17], and helps get parents active, a demographic plagued by lower PA rates compared to comparable age-matched adults without children [18, 19].

Intervention research targeting parent-child co-activity has shown positive effects [20, 21], which highlights the promise of family-based intervention. However, this is balanced by several reviews indicating interventions targeting parent-child co-activity have largely lacked success [22–25]. This underscores a clear need to continue to hone theory behind parent-child co-activity to facilitate reliable success. The reason for these negligible findings may be because the dominant approach is educating parents on the benefits of PA [13], which does not appear to be a driver for behaviour change, likely because the benefits of PA are already well-recognized among parents [14, 26–29]. Furthermore, a recent study suggests that most parents have positive intentions to support child PA, yet few follow through on enacting this support [30]. Clearly, existing intervention targets for parental support have not been effective at bridging the intention-behaviour gap. Taken together, there is a need to focus on how intentions to support child PA (specifically through parent-child co-activity) can be translated into behaviour and sustained over the long term.

In order to bridge the intention-behaviour gap to support child PA through co-activity and foster behavioural follow through, theoretical approaches for behaviour change that move beyond intentional constructs are needed. Several alternate models propose that self-regulation strategies such as planning are integral (e.g., [31–33]). This type of approach has been applied to family-based interventions and has shown success in the short term [13]. However, planning may be difficult for parents to sustain over time, risking a failure to maintain parental support of child PA. As an example, a recent study comparing a family-based planning condition to an education only control condition found significantly increased child PA at 6 weeks but the effect waned over time, suggesting additional strategies are needed for PA maintenance [34]. Of particular importance, the process evaluation of this study indicated that several parents rejected the planning approach, citing that it was exhausting unto itself and thus unsustainable.

One approach that may assist in converting intentions into lasting parental support (in the form of parent-child co-activity) is the Multi-Process Action Control (M-PAC) framework [35, 36]. M-PAC proposes that self-regulatory tactics, such as planning, assist with the translation of intentions during the adoption of a behaviour, but maintenance is determined, in part, by reflexive components such as habit. This approach suggests that developing habits over time can reduce the need for conscious motivation and self-regulation, thereby facilitating sustained behaviour. Habits operate through stimulus-response bonds which automatically instigate a behaviour [37], and are thought to contribute to physical activity through repeated consistency in behavioural practices, salient cues linked to initiation, and affectively rewarding behaviour [38–40]. Extant research in the PA domain indicates interventions designed to promote habit strength through prompts, cues, context-dependent repetition and consistent practice may have promise in promoting PA [37, 41–44], likely because habits contribute to PA maintenance somewhat separately from intention (or goals) [41, 45]. Indeed, a recent study applying M-PAC to parental support for child PA found habit to be the largest independent correlate of behavioural follow-through [30], indicating that interventions that promote habit strength are warranted.

### Study objectives and hypotheses

The primary objective of this study is to investigate the effects of a theory-based intervention targeting parent-child co-activity to promote child MVPA (primary outcome) across 6 months. Namely, the effect of a *habit formation* (context-dependent repetition from prompts and cues, consistent practice) and planning and education condition (action planning, coping planning, and information on benefits), will be compared against a *planning* and education condition (action planning, coping planning and information on benefits) and a standard *education* condition (information on benefits) on child PA. Consistent with previous research [30, 46], it is hypothesized that the habit formation intervention targeting parent-child co-activity will yield greater increases in co-activity and subsequently influence objectively measured child MVPA at 6 months (primary end-point) compared to those in the planning and education conditions. Further, based on extant research on the efficacy of planning interventions among families [34], we predict that children in the planning condition will show greater increases in MVPA compared to the education-only group because planning aids PA adoption, however these initially efficacious effects will wane over time due exhausting nature of constant volitional planning and the lack of strategies to assist with maintenance.

The secondary objective will be to explore group differences among behavioural outcomes using a mediation model. We hypothesize that child PA will be influenced by parent-child co-activity, which will be a result of PA support from parents in the form of co-participation. Higher PA support (co-participation) is expected to come from those in the habit formation condition, followed by self-regulatory planning compared to education. Thus, it is hypothesized that the covariance of the assigned condition (habit formation, planning, and education) will be explained by parental co-activity habit strength through the use of context repetition/cues and consistency/repeated action (i.e., manipulation check).

Tertiary objectives include evaluating both self-reported and objectively measured physical activity for parents as well as parent-child co-activity (via accelerometry using a Bluetooth enabled proximity tagging feature). It is hypothesized that parents in the habit formation condition will show higher light and moderate intensity PA compared to the other conditions due to consistent performance of co-activity with their children.

Additional parental factors such as quality of life, parental competence, and family functioning will also be examined. We hypothesize that conditions that increase PA will show commensurate increases in these factors. Finally, exploratory analyses will focus on seasonal, or gender differences across primary outcomes by assigned condition [47, 48].

## Methods

**Fig. 1:**
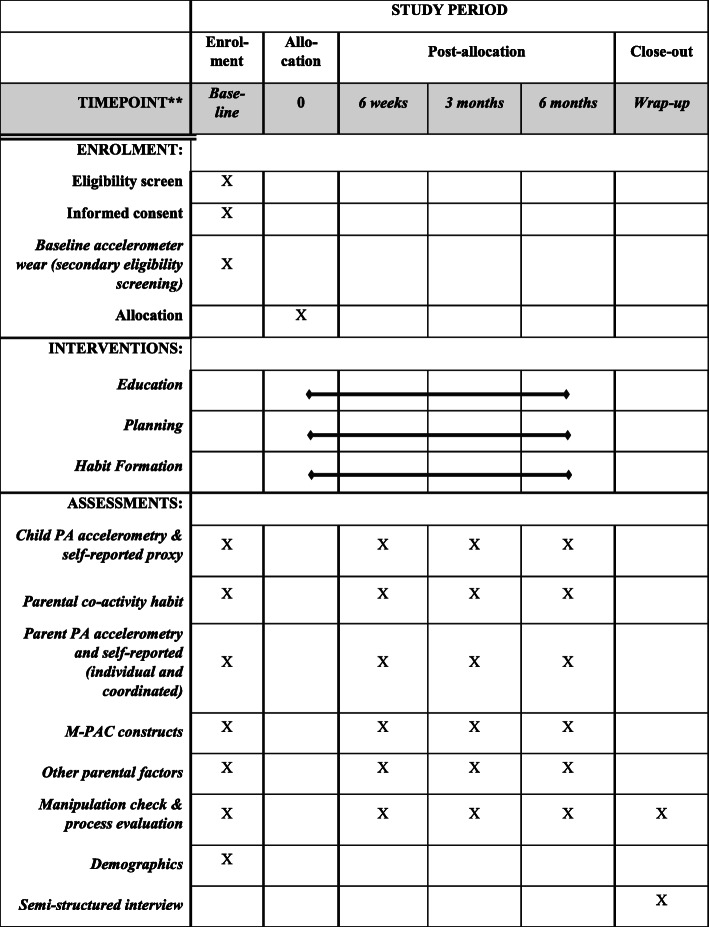
SPIRIT 2013 Schedule of enrolment, interventions, and assessments

This study is approved by the University of Victoria Human Research Ethics Board (HREB), reference number BC16–231. The reporting of the trial follows the Standard Protocol Items: Recommendations for Interventional Trials (SPIRIT) guidelines [49] and will harmonize with the Consolidated Standards of Reporting Trials (CONSORT) guidelines [50]. For SPIRIT information, a diagram is included (see Fig. 1). The trial was prospectively registered with the Clinical Trials Registry maintained by the National Library of Medicine at the National Institutes of Health (ClinicalTrials.gov) Trial ID NCT03055871.

### Design

This study is a randomized trial with a single-blinded, three-arm parallel design. Upon the completion of baseline measures (questionnaire, accelerometer wear) participants are assigned to one of three groups: 1) standard co-activity education (education condition); 2) co-activity education and planning (planning condition); 3) co-activity education, planning, and habit formation (habit formation condition). The main outcome measure of child MVPA (dependent variable) will be assessed at the primary end point of the trial (6 months) with secondary assessments time points at 6 weeks and 3 months.

### Participants and eligibility

To be considered eligible, families must reside in the greater Victoria region, in British Columbia, Canada. Both single and common-law/married adults are eligible so long as they have at least one child between ages of three and 5 years. This age range was informed by a literature review [51] and survey data suggesting that children aged 5 years or younger engage in sporadic PA that is not amenable to formal sport programs and thus parent-facilitated co-activity is critical. Age range was also delimited on pragmatic grounds: school-aged children often start structured PA/sport and engage in peer play which does not lend itself to parent-facilitated co-activity. In cases where more than one child is eligible, a target child will be randomly chosen but all willing children will be included in the study. It is required that families are safe to engage in moderate intensity physical activity (assessed via the Get Active Questionnaire [52]. Those individuals who are not able to participate in moderate intensity physical activity will be excluded for safety reasons. Finally, families are only included if parent-declared child PA is less than recommended by the Canadian 24 Hour Movement Guidelines [53]. Because inactive children are the true target of such an intervention, it is important to delimit participants to children not meeting PA guidelines and it follows families are also screened via baseline accelerometry results as a secondary assurance. Families with designated children who meet the Canadian guidelines are subsequently excluded from the trial. Target parent(s) PA is not an eligibility criterion because increases in parent PA is not a primary outcome.

### Recruitment procedure

The recruitment methods of this study follow a previously refined recruitment strategy similar to published protocols conducted within the same laboratory [54, 55]. The primary means of recruitment are targeted advertisements on online interest sites (blogs, parent resource websites, and classifieds) and social media platforms (Facebook, Instagram). For social media posts, the visual and written content are geared toward families. Posts are also targeted to a local parent audience by selecting restrictions for age range, keywords, and interests. A small fee applies these demographic filters and allows the ad to run for about 7 days. Posts are also manually shared to relevant Facebook groups (e.g., parent groups). In addition, posters are regularly placed around the Greater Victoria region at locations such as daycares and preschools, community centres, recreation centres, local coffee shops, and health care centres. Twice a month, a designated Recruitment Officer also offers pamphlets and study information through stalls at family-oriented events such as community markets. In order to ensure a diverse study population, facilities from each region of the city were systematically selected and contacted for recruitment. Recruitment also takes place through word of mouth, whereby current participants refer the study to others by passing on information. Incentives for participation include honoraria at each respective assessment to compensate participants for their time ($25 per family, increasing by $5 increments at each assessment for a total $130 across the study).

Recruitment is rolling across the duration of the study. Families interested in partaking are invited to contact the researchers via email or phone. Upon receipt of inquiries, potential participants are sent additional study details over email and are invited to schedule a phone call. Next, a Recruitment Officer formally screens potential participants over the phone. If they are deemed eligible, they are scheduled for a baseline assessment. Written informed consent is obtained from parents and verbal assent is obtained from children before study enrolment.

### Randomization

Simple randomization is done by the Project Coordinator using computer-generated random numbers in an Excel spreadsheet. Allocation is concealed from the enrolment and assessment team by the Project Coordinator. Upon completion of baseline measures, condition allocation is communicated to the intervention delivery team so as to allow for the correct delivery of materials. Participants are blind to their condition until study completion, at which point they are notified of their allocation by the research team and provided with any additional materials they did not originally receive.

### Procedures and protocol

Once deemed eligible and enrolled, participants are scheduled for a baseline assessment. At this initial meeting, parental consent and child assent are obtained, participating parent(s) are asked to complete a questionnaire, and the research team sends accelerometers home for the participating parent(s) and child (ren) for a week of wear. Upon completion of this objective PA measure, child MVPA is checked against PA guidelines [56] and a secondary screening procedure excludes those families with children meeting guidelines from the trial. Families are then randomized into one of three intervention conditions and a Research Assistant delivers the appropriate intervention. Condition allocation will not require alteration of usual care pathways, and these will continue for all trial arms.

Next, the Project Coordinator schedules all families for “booster” sessions at 6 weeks and 3 months. These sessions involve a house visit by the Research Assistant to discuss any successes, progress, challenges, or setbacks. To encourage adherence to the relevant intervention information, the Research Assistant recaps the relevant intervention materials and facilitates family problem solving based on the family’s experience (see Table 1 for Behaviour Change Techniques per condition). Standardized interviewing techniques are employed [57]. For example, if the Research Assistant learns the family has not been planning their physical activity or employing cues for habit formation, they would discuss how the family may implement these strategies. At these sessions the research assistant also leaves accelerometers and asks the family to complete the online questionnaire at their convenience so long as it is completed within the week.

**Table 1:** Description of intervention components and associated behaviour change techniques

Intervention condition	Resources included in booklet	Behaviour change technique label	BCT Number
Education	1. Canadian 24-h Movement Guidelines 2. Benefits of co-activity	Instruction on how to perform a behaviour	4.1
		Information about health consequences	5.1
		Salience of consequences	5.2
		Information about social and environmental consequences	5.3
		Information about emotional consequences	5.6
Planning (+ education)	1. Explanation of goal setting and SMART goal materials 2. Definition of self-monitoring and worksheets for tracking co-activity and outcomes 3. Rationale for planning and co-activity planning worksheets 4. Information on the importance of enjoyment and brainstorming worksheets on fun new co-activities, stimulating environments, and reward structures	Goal setting (behaviour)	1.1
		Problem solving	1.2
		Action planning	1.4
		Self-monitoring of behaviour	2.3
		Self-monitoring of outcome(s) of behaviour	2.4
		Social support (practical)	3.2
		Social support (emotional)	3.3
		Non-specific reward	10.3
		Self-incentive	10.7
		Restructure physical environment	12.1
		Restructure social environment	12.2
Habit (+ education and planning)	1. 1. 1. Habit formation information 2. Examples of habits 3. Cues and anchoring 4. Brainstorming existing routines for tagging co-activity	Prompts/Cues	7.1
		Behavioural practice/rehearsal	8.1
		Habit formation	8.3

*Note.* Behaviour change techniques are coded according to the Behaviour Change Technique Taxonomy Version 1 [58]

Finally, at the six-month mark, accelerometers are left with the family and a final online questionnaire is sent via email. After the week of accelerometer wear, the family is asked to return to the lab for a final meeting and exit interview which qualitatively explores evaluations of the impact of the intervention. Semi-structured interviews are employed to examine both content fidelity and process fidelity of the intervention delivery of the trial.

### Intervention

The interventions are conducted in-person with the family and are delivered by a research assistant. To ensure implementation fidelity, research assistants receive in-depth training on the intervention’s concepts, techniques, and conduct. Interventions for each respective condition involve particular take away materials for the family to employ on their own time. Specifically, a specialized hard-copy physical activity workbook (designed for families and geared towards parents) serves as a template for discussion for all three conditions and is left with the family to fill in and refer to over the course of the study. The Research Assistant explains the concepts and associated worksheets and relays a clear expectation that the workbook is to be completed and used over the course of the study. The workbooks feature colourful imagery and graphic design showcasing family physical activity. The material integrates established Behaviour Change Techniques (BCTs) that are consistent with the M-PAC framework [35]. As per Michie and colleagues’ [58] taxonomy, a complete breakdown of the BCTs employed across conditions is available in Table 1.

The materials are based on previous family-based physical activity interventions targeting self-regulatory processes [34, 59, 60]. Notably, the interventions build upon a recent family physical activity planning randomized trial [34] and advance prior work from a habit formation pilot trial [38]. The habit condition is of key interest. The habit formation condition aims to bring about automatic initiation (or ‘instigation’ [61] of co-activity, such that encountering a cue is sufficient to prompt engagement in co-activity, rather than any available behavioural alternatives, in the absence of any deliberative decision-making process.

### Education condition

The information presented to the education condition includes the Canadian 24 Hour Movement Guidelines, which recommend 180 min of PA at any intensity spread throughout the day with at least 60 min of energetic play [53]. Additionally, facts surrounding the health outcomes of physical activity are provided, including the physical, psychological, social and emotional benefits (BCTs 5.1, 5.2, 5.3, 5.6). An explanation of MVPA and ideas for co-activities are presented with structured and unstructured examples cited (BCT 4.1). Finally, a brainstorming activity is included about potential benefits arising out of co-activity as well as current and possible co-activities.

### Education and planning condition

Those in the planning condition receive all the educational material outlined above (guidelines, benefits, and co-activity ideas/instruction). In addition, the planning condition is presented with various self-regulatory strategies to facilitate problem solving (BCT 1.2) and aid co-activity adoption. Strategies covered include goal setting (how to set SMART goals; BCT 1.1), action planning (explanation and worksheets incorporating where, when, and how to engage in co-activity; BCT 1.4), and self-monitoring (definition and journaling and tracking worksheets to log participation and outcomes; BCT 2.3, 2.4). Furthermore, participants are instructed on the importance of affective attitudes and rewards (BCT 10.3, 10.7) then asked to brainstorm fun new modes of co-activity, enjoyable environments, and potential rewards for being active together, which subsequently allows for the identification of the necessary social and environmental supports (BCT construct 3.2, 3.3, 12.1, 12.2; see Table 1 for complete breakdown of intervention component and BCTs for the planning condition).

### Education, planning, and habit formation condition

The habit formation condition receives the same content as the education condition, plus the regulatory strategies from the planning condition, as well as material on how to develop habits for initiating co-activity. The materials are based on habit formation research [39], informed by a successful pilot study [38], and adapted for families. The habit formation section includes a discussion of what constitutes a habit with non-domain specific examples (night-time routine, morning commute). A fundamental element of the habit section is the idea of utilizing context-dependent repetition (BCT 8.1, 8.3), which includes recommendations on how to nurture repetition during the formation of habits. Namely, a passage on routines and a subsequent brainstorming activity encourages the elicitation of existing routines relevant to the family (e.g., pizza night every Friday). It is explained that tagging PA to these existing routines is an effective way to support repetition. Indeed, this approach has shown efficacy in habit formation in other behavioural domains [62], presumably because it optimizes context-dependent performance by encouraging consistent behavioural patterns, in effect fulfilling the requirements for habit formation. Next, cues (BCT 7.1) are introduced as a factor that prompt behaviour, thereby supporting habit formation. The various forms of habit are then discussed (temporal, social, mood, and visual) with relevant examples. The need for cues to precede the activity and otherwise be rarely present is highlighted. The key to this concept is stimulus-behaviour linkage. For example, a swimsuit bag set by the door to prompt an afternoon family swim might serve as an effective cue as it is highly likely it will activate the behaviour at the opportune moment. Conversely, an ever-present sign by the door would reduce the salience of the cue and in result minimize its potential for activating the desired behaviour. For instance, if one is walking by the door without engaging in activity, there is a theoretical weakening of the link to automatic action over time. It might serve as a reminder for cognitive action, but the cue will not have a strong association if it is not performed with a repeated behavior very often. The next important recommendation is to have consistency in co-activity practices (in the form of protected time for various activities), in order to support instigation habits. Finally, a poster-style summary outlining ten evidence-based tips for translating physical activity intentions into action is provided. The Research Assistant clearly communicates that the workbook should be completed, and the habit formation ideas should be implemented.

### Outcome measures

#### Primary outcome measures

The primary outcome of child MVPA will be assessed with the Actigraph wGT3X-BT Activity Monitor using validated seven-day accelerometry methods for preschoolers. Measurements will take place at baseline, 6 weeks, 3 months, and 6 months (primary endpoint of interest) and MVPA will be evaluated as change from baseline. Currently, the analysis plan is to use the established cut-points and accelerometer best practices outlined below, however the research team recognizes that accelerometer analysis in an ever-changing discipline, therefore we will be responsive to these advances by using the most validated cut-points at the time of study closure. At present, it is anticipated that the MVPA of children will be determined using the cut-points for young children recommended by Janssen and colleagues [63]. These cut-points define moderate to vigorous activity as ≥420 counts per 15 s (≥1680 counts per minute (CPM)). These cut-points have shown fair to excellent validity in preschoolers when compared to direct observation [63].

Children are instructed to wear the device during waking hours for a minimum of 10 h a day for 7 consecutive days (5 weekdays, and 2 weekend days). It is secured by an elastic band and worn around the waist above the right hip. Because the devices are not waterproof, participants are advised to remove them for any water-based activities. In addition, participants are provided with a logbook and parents are asked to note any removals for water activities, record unusual circumstances (e.g., changes to routine such as illness), and make notes with particulars such as structured activity.

To initialize and download the accelerometers as well as analyze the data, ActiLife software version 6.11.9 [64] is used. The devices are initialized to collect data at a 30 Hz sample rate and are downloaded into 15-s epochs in order to capture the sporadic nature of movement in young children and based on common practice for preschooler accelerometry [65, 66]. Data will be included for analysis if children have at least 4 days of valid wear time per week [67–69]. For a day to be considered valid there will need to be a minimum of 300 min (6 h) during waking hours [70]. Non-wear time is defined as ≥20 min of consecutive zero counts or the equivalent of ≥80 consecutive 15-s intervals of zero counts [69, 71]. It is assumed that this non-wear time algorithm captures daytime naps.

### Secondary outcome measures

#### Parent-reported proxy child physical activity

In addition to objective PA assessments for children described earlier, a secondary outcome includes child PA measured via parent proxy self-report. Specifically, parents are asked to report their child’s physical activity as well as family-co-activity (both structured and unstructured) at all measurement occasions (baseline, 6 weeks, 3 months, and 6 months) using an adapted Godin Leisure-Time Exercise Questionnaires [72] modified for co-activity [60, 73]. This instrument is included in addition to objectively measured PA because the measurement methods are not equivalent but can be complementary: self-report is distinct as it allows for the assessment of volitional PA [74] and a more focused report of PA specifically as a family. While parent proxy measures are certainly less accurate than accelerometry, this measure has shown sensitivity to change in intervention and predictive validity in observational data [73].

#### Parental co-activity habit

At all measurement occasions (baseline, 6 weeks, 3 months, and 6 months), parental co-activity habits for child PA will be measured with an adapted self-reported habit strength index [75] which includes the self-reported behavioral automaticity index subscale [76], both of which have demonstrated excellent reliability and validity in self-reported and objective PA assessment [77]. These measures have been used in prior research assessing habit of parental support within M-PAC [30]. Therefore, the scale follows the same form but was slightly modified for co-activity and thus the 12 items are as follows: “regular physical activity with my child is something I do... frequently, automatically, etc.” answered on a five-point Likert scale.

### Tertiary outcome measures

#### Parental physical activity

The tertiary outcome of parental PA is measured both by self-report and seven-day accelerometry at all assessment occasions (baseline, 6 weeks, 3 months and 6 months). Accelerometry will be measured in a similar fashion to the methods outlined above. Namely, parents are instructed to wear the Actigraph wGT3X-BT for 7 days a week for a minimum of 10 h a day, remove it at night and for water-based activities, and note the details of the wear in a logbook. Activity will be assessed by measuring several variables, including duration (total minutes worn, total movement counts/day, total minutes of sedentary, light, moderate-vigorous day), frequency (bouts of sedentary, light moderate-vigorous/day), and intensity. To calculate these variables data will be collected in 10 s intervals and reintegrated into 60 s epochs to align with the methods of Troiano et al., [78]. Non-wear time will be determined using the algorithm from Troiano et al., [78] and will be subtracted from total wear time. As per recommended best practice [79, 80], parents need at least 4 days with 10 h of valid wear per week. Accelerometers have demonstrated reliability and validity when worn for ≥4 days [81–83]. The MVPA of adults will be evaluated as change from baseline and determined using frequently applied cut points [66] validated for adults [78] which classify MVPA as 2020 counts per minutes (CPM) and above. Again, the research team is mindful of the rapidly evolving nature of accelerometry analysis and therefore we will be responsive to the most validated cut-point norms in the field at the time of study closure.

As a secondary self-reported parent PA outcome measure, the Godin Leisure-Time Exercise Questionnaire (GLTEQ), is employed [72, 84] which assesses the frequency and duration of mild, moderate, and strenuous activity performed during free time in a typical week in an open-ended manner. A total weekly MVPA score will be calculated by multiplying the frequency by the duration.

#### Parent-child co-activity

Parent-child co-activity is assessed at all measurement occasions via self-report (with a modified GLTEQ; 71) and objectively via accelerometry. Accelerometry will be measured as discussed in the outcomes above and tagged with the child to assess co-activity. A relatively new feature of the accelerometer is the ability to determine the presence of other nearby people (i.e., parent or child), who are also wearing accelerometers. This Bluetooth-enabled feature allows for the objective assessment of co-activity between parent and child. In this method, “beacon” devices broadcast their serial number and “receiver” devices are set to search for the signal once per minute. The receivers then store a log of proximity detection information, recorded as a received signal strength indicatior (RSSI). In the present study, the child’s monitor is set as the “receiver” and the parent’s monitor (secondary outcome) is set as the “beacon”. Every 60 s where the monitors are in close proximity will be “tagged” and recorded in the memory of the child’s monitor, which will be later linked to time-stamped data of the parent. According to ActiGraph [85], accelerometers communicate via Bluetooth when in close proximity indoors, which is a maximum of 10–20 m, and close proximity outdoors, which is a maximum of 100 m. One study examining Bluetooth-enabled accelerometers found acceptable validity for determining whether parents and children are in close proximity [86]. Another study examining the validity and reliability of the feature found proximity detection with Bluetooth-enabled accelerometers was reliable in controlled settings and had reasonable rates of detection during free-living conditions albeit only when in close proximity [87].

#### M-PAC constructs for parent-child co-activity

Beyond habit, the additional M-PAC constructs are framed for parent-child co-activity. These measures include motivational (instrumental attitude, affective attitude, and perceived capability), regulatory (planning), and reflexive (automaticity, identity) elements. Intention is assessed using Courneya’s [88] recommendation for open-scaled measurement. Attitudes toward engaging in parent-child co-activity are measured with items covering both affective and instrumental components [89, 90], and items related to ability for parent-child co-activity assess perceived capability [91, 92]. An instrument adapted from Sniehotta and colleagues [93] assesses behavioural regulation. Lastly, items adapted from Anderson and Cychosz [94] measure identity. All of these instruments have previous application in various populations and have demonstrated previous strong predictive validity and internal consistency evidence scores among adults [95–97], and with parental physical activity support. Finally, the measures have shown some prior validity evidence for family-based physical activity as well as personal physical activity [30, 73].

#### Other parental factors

Several additional parental factors are assessed at all measurement points (baseline, 6 weeks, 3 months, and 6 months). Namely, instruments that have shown adequate validity and reliability in prior test scores measure the following: quality of life is determined via the 12-item Short-Form Health Survey [98]; parental competence is assessed with the Parenting Sense of Competence Scale [99]; and family functioning is assessed through the Family Environment Scale [100].

#### Demographics

A section of the baseline questionnaire assesses various demographic characteristics including gender, age, ethnicity, level of education, marital status, employment information, household income, and health background.

#### Manipulation check outcomes

To examine the manipulation check outcomes, items assessing parental self-report of the utilization of context-dependent repetition/cues and consistency/repeated action are used [38]. These measures take place at all measurement time points. For the measure of context-dependent repetition/cue utilization, parents are presented with the phrase “each time I am physically active with my child” and are asked to rank six statements on a seven-point scale from “not true at all” to “very true”. The statements incorporate temporal (‘it was the same time of day’), visual (‘I was in the same place’), social (‘I was around the same people’), and mood (‘I was in the same mood’) aspects. The single item consistency of practice measure asks parents “how consistently are you physically active with your child at the same time each day?” with answers on a five-point scale ranging from not consistent to very consistent. This measure assists with understanding repeated action and is therefore a reasonable assessment of the utilization of key habit formation techniques.

Additionally, parents complete a short process evaluation of the intervention in the six-month questionnaire. First, a brief questionnaire assesses use of the intervention materials and satisfaction with the study [101]. Finally, semi-structured interviews pertaining to types and frequency of co-activity, barriers, outcomes, intervention material use and study satisfaction are conducted at the end of the trial, in order to gain rich, in-depth program evaluation data that has proved useful in prior trials [34]. These interviews will also incorporate simple quantitative questions regarding the use and ease of the intervention materials.

### Analysis strategy

For each outcome, the pattern of missing data will be evaluated to determine an appropriate analytical strategy [102], after which the normality of all variables will be examined to determine whether transformations will be required. The first set of analyses will make preliminary demographic comparisons among adherers to the study versus dropouts to determine the representativeness of the sample. Next, to address the primary objective (i.e., to determine whether the minutes / day of MVPA change over time similarly for all three conditions, hierarchical linear modeling [103] will be used. Specifically, Level-1 of the model will include an intercept (i.e., the baseline PA) and slope (i.e., to examine potential change over the 4 assessment periods) that will be predicted by Level-2 covariates (i.e., demographics). Additionally, dummy variables will be created for condition (Habit formation group: 1 = yes or 0 = no; Planning group: 1 = yes or 0 = no; Education control: 1 = yes or 0 = No) at Level-2 with the Habit Formation and Planning group variables being added to the model to predict the intercept and slope at Level-1. In doing so, the control group is compared against the other two groups to determine if baseline MVPA is similar across conditions and whether the change in MVPA is similar across conditions. Follow-up analyses will be conducted for the Habit Formation vs. Planning group comparison. To address the secondary objective (i.e., to determine whether the change in the underlying motives explain the potential change in MVPA during the intervention similarly for all 3 groups), a time varying covariate mediation analysis will be used [104]. Briefly, the analyses needed to establish mediation will treat the underlying motives as time varying covariates at Level-1 of the model. Then, the dummy coded condition variables will be entered at Level-2 to determine if the Level-1 motives mediate the condition / MVPA relationship. Finally, to determine whether there is a seasonal, intergenerational, or gender difference across the primary and secondary outcomes (i.e., to address the tertiary objectives), each variable will be entered into the various models at Level-2 to predict the intercepts and slopes at Level-1. Doing so will determine if they impact the change in the various outcomes across time.

Qualitative data analysis will be conducted by research assistants independent from the intervention activities and will be overseen by the principal investigator. This component of intervention evaluation will draw from a qualitative social constructionist perspective [105] to understand in the parents’ own words, the beneficial features and any problematic components of the intervention. Data collected via the semi-structured interviews will be analysed through use of inductive content analytic procedures [106], facilitated by the NVivo software program. Themes will be identified that correspond to the strengths and limitations of the intervention program. The response themes will also be linked at the individual level, so post-hoc assessment of successful and unsuccessful interventions can be examined by these responses.

### Justification of sample size

G-Power (Version 3.1.9.2) was used to calculate sample size. With 4 repeated assessments, 3 groups, a power of .90, an alpha of .05, effect sizes ranging from .25 to .30, and an anticipated attrition rate of 15% [34], a minimum of 165 families with a goal of 240 families (i.e., 55–80 families per condition) are needed to show a significant difference in physical activity accelerometry (minutes of MVPA primary outcome) by condition over time. The effect sizes represent the findings from our prior intervention research comparing planning to education with this demographic [34] and considering our pilot study on habit formation [42], yet it is clearly in the clinically meaningful range for the detection of differences between the planning and habit formation conditions [107, 108]. However, detection is dependent on the performance of the habit versus planning conditions which is largely unknown.

### Oversight, data monitoring, ethics, dissemination

#### Data management and confidentiality

Confidentiality procedures are outlined in the consent form and explained by the Research Assistant at the baseline assessment during the informed consent procedure. Participants are provided with unique identifiers and assured that data will be published as group data. Contact information is required but carefully protected: hard copies of any documentation are kept in a locked and secure environment at the University of Victoria. Any data or personal information stored on computers is kept on a secure server. Questionnaire data are stored on SurveyMonkey servers in Canada. If participants withdraw, they may choose whether their data will be used or destroyed. The research team determined a formal data monitoring committee was not necessary. In lieu, the Project Coordinator provides monthly reports on trial progress, participant numbers, and data quality to the Principal Investigator. Project oversight is the responsibility of the Principal Investigator, and therefore the decision to terminate the trial rests with them. Finally, access to the final dataset will be made to the Principal Investigator.

#### Research ethics processes and monitoring

If modifications or amendments to the protocol are required, the Project Coordinator will submit the necessary paperwork to HREB at the University of Victoria. Upon approval, the appropriate updates will be made to the trial registration on the Clinical Trials Registry. There are no anticipated harms that will result from participation. In the case of unintended effects or adverse events, participants are provided with contact information and are instructed to inform the Project Coordinator, the Primary Investigator, or HREB. In the instance of any such events, the research team is trained in documenting and reporting procedures and the safety of all parties is prioritized at all times.

#### Dissemination plans

Trial results will be widely disseminated by way of knowledge exchange activities, such as presentations at academic conferences and publications in relevant journals. Eligibility for authorship in subsequent publications is available to all those who have contributed to the design and protocol of the trial. Findings from this trial could have important implications for public health. For example, results could inform initiatives to improve PA among families with young children. In line with HREB approval, the participant data set will not be accessible for the public. At present, there are no arrangements to make the statistical code available to the public. Results from the trial will be communicated to participants.

## Discussion

**Fig. 2:**
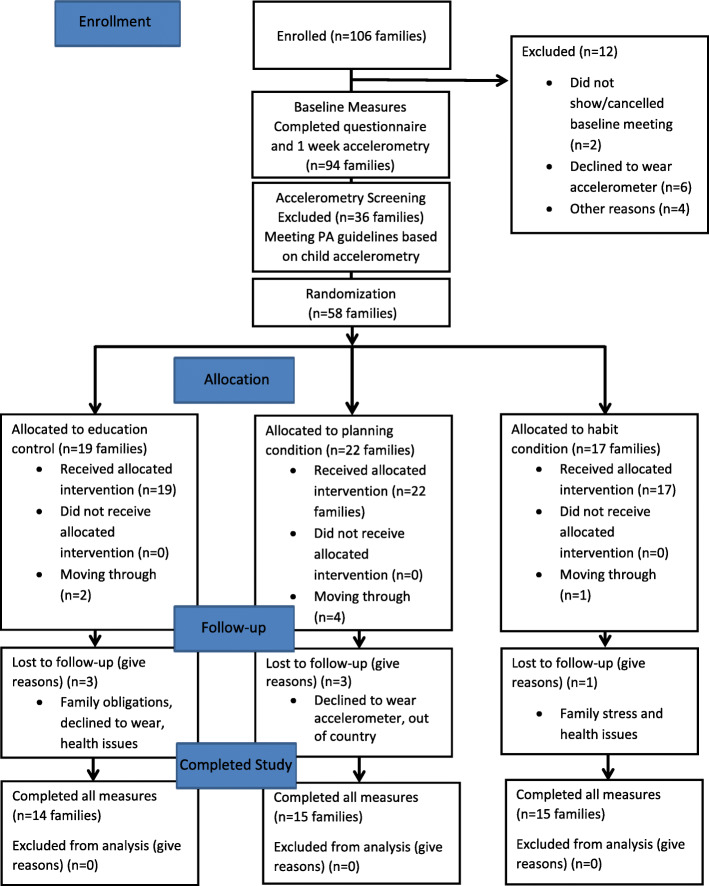
Participant Flow Diagram (Clinical Trial Registration Number: NCT03055871)

To date, we have obtained ethical approval, registered the trial, and have recruited 106 families from the Greater Victoria region. Ethical approval was received from the University of Victoria Human Research Ethics Board. We currently anticipate recruitment will be complete by August 2022. From the 106 parents assessed for eligibility, 94 have completed all of the baseline measures, 49 have completed the six-week measures, and 47 have completed the 3 month measures, and 44 have completed the 6 month measures and exit interview concluding the study (see Fig. 2 for Participant Flow Diagram). The study is ongoing and data collection will continue through 2021 (with the possibility of extending into 2022).

This protocol describes the implementation of a randomized trial investigating the efficacy of planning and habit interventions aimed at increasing parent-child co-activity. This research is conceptually guided by the M-PAC framework [35]. One anticipated challenge may be the differentiation between those who develop habits naturally as a consequence of repeated planning from those who develop habit from direct intervention in our study. Specifically, M-PAC outlines that habits can form from as a natural extension of self-regulatory planning, under conditions where context repetition is observed over time [109], and this has also been noted by other habit researchers (e.g., [39, 43]. We expect that our direct intervention condition would facilitate for faster changes in habit strength in this case, but we will explore this possibility of direct and indirect habit formation. Our intervention success may also be challenged by the length of the trial. While 6 months duration has been sufficient to produce changes in PA habit strength among adults [38], the dynamics involved in a family system with multiple stakeholders in the intervention may be more challenging when attempting to create the context repetition stability [110] necessary to form a habit. Our qualitative feedback from the intervention will be instrumental in understanding the complexities of this approach in a family system.

Findings from this trial could contribute to the understanding of strategies to increase child PA through targeting parent-child co-activity. This information could prove useful for public health initiatives aimed at improving PA participation among children and families.

## Data Availability

The datasets generated and/or analysed during the current study are not publicly available as per ethical approval from HREB at the University of Victoria, which stipulates that the data will not accessed or analyzed by others.

## Abbreviations

PA: Physical activity
RCT: Randomized controlled trial
HREB: Human research ethics board at the university of Victoria
